# Cell surface heparan sulfate proteoglycans control adhesion and invasion of breast carcinoma cells

**DOI:** 10.1186/s12943-014-0279-8

**Published:** 2015-01-27

**Authors:** Hooi Ching Lim, Hinke AB Multhaupt, John R Couchman

**Affiliations:** Department of Biomedical Sciences and Biotech Research & Innovation Center, University of Copenhagen, Biocenter, Ole Maaløes Vej 5, 2200 Copenhagen N, Denmark; Current address: Stem Cell Center, Lund University, Lund, Sweden

**Keywords:** Cytoskeleton, Adhesion, Syndecan, Caveolin, Tumor cells, Tissue microarray

## Abstract

**Background:**

Cell surface proteoglycans interact with numerous regulators of cell behavior through their glycosaminoglycan chains. The syndecan family of transmembrane proteoglycans are virtually ubiquitous cell surface receptors that are implicated in the progression of some tumors, including breast carcinoma. This may derive from their regulation of cell adhesion, but roles for specific syndecans are unresolved.

**Methods:**

The MDA-MB231 human breast carcinoma cell line was exposed to exogenous glycosaminoglycans and changes in cell behavior monitored by western blotting, immunocytochemistry, invasion and collagen degradation assays. Selected receptors including PAR-1 and syndecans were depleted by siRNA treatments to assess cell morphology and behavior. Immunohistochemistry for syndecan-2 and its interacting partner, caveolin-2 was performed on human breast tumor tissue arrays. Two-tailed paired t-test and one-way ANOVA with Tukey’s post-hoc test were used in the analysis of data.

**Results:**

MDA-MB231 cells were shown to be highly sensitive to exogenous heparan sulfate or heparin, promoting increased spreading, focal adhesion and adherens junction formation with concomitantly reduced invasion and matrix degradation. The molecular basis for this effect was revealed to have two components. First, thrombin inhibition contributed to enhanced cell adhesion and reduced invasion. Second, a specific loss of cell surface syndecan-2 was noted. The ensuing junction formation was dependent on syndecan-4, whose role in promoting actin cytoskeletal organization is known. Syndecan-2 interacts with, and may regulate, caveolin-2. Depletion of either molecule had the same adhesion-promoting influence, along with reduced invasion, confirming a role for this complex in maintaining the invasive phenotype of mammary carcinoma cells. Finally, both syndecan-2 and caveolin-2 were upregulated in tissue arrays from breast cancer patients compared to normal mammary tissue. Moreover their expression levels were correlated in triple negative breast cancers.

**Conclusion:**

Cell surface proteoglycans, notably syndecan-2, may be important regulators of breast carcinoma progression through regulation of cytoskeleton, cell adhesion and invasion.

## Introduction

Metastasis is a multistep process involving dissemination of cancer cells from a primary tumour to distant organs [1]. During metastasis from solid tumours, cells change their adhesion status to facilitate migration through basement membrane and extracellular matrix, enter the bloodstream or lymphatics, extravasate into distant organs and eventually proliferate to form metastases [2]. Understanding the molecular basis of tumour cell motility and invasion is crucial to identify attractive targets for potential therapeutic intervention.

In mammals, syndecans are a four-member family of cell surface heparan sulfate proteoglycans that are well placed to be important regulators of cell migration and tumor progression. While expression of each syndecan has some tissue- and cell type- specificity, they can bind to a wide range of proteins including growth factors, cytokines, chemokines, morphogens, extracellular matrix proteins, proteinases and proteinase inhibitors through their heparan sulfate chains [3,4]. Syndecans have an ability to regulate cell motility, cell-cell and cell-extracellular matrix adhesion through signaling to the actin cytoskeleton, with which all syndecans can interact [5,6]. Additionally, the core protein of syndecans can directly or indirectly promote integrin-mediated adhesion and integrin turnover [7-11]. Although not possessing intrinsic kinase activity, the syndecan proteoglycans can nevertheless signal through their cytoplasmic domains, mediated by specific binding partners, e.g. protein kinase Cα in the case of syndecan-4 [12-14].

In tumors, syndecan expression is frequently altered during malignant transformation and may contribute to tumor progression [15-18]. For example, syndecan-1 expression and its shedding from the cell surface appear to relate directly to myeloma progression [15,19]. Syndecan-4 may play differing roles in modulating tumor cell invasiveness depending on the cancer type. In breast carcinoma, syndecan-1 can promote cell spreading and adhesion to extracellular matrix with subsequent inhibition of cell invasion [20]. We showed that syndecan-4 expression in human breast carcinoma tissues correlates with positive estrogen and progesterone receptor status therefore a good prognosis [21]. Conversely, syndecan-1 expression in breast carcinoma is an indicator of poor prognosis, particularly where it is stromal [21,22]. Syndecan-2 expression is upregulated in colon cancer, pancreatic cancer, melanoma and fibrosarcoma where it enhances cell adhesion, proliferation and migration in cancer cells, suggesting that it is important in promoting tumor progression [23-26]. However, while emerging evidence suggests that syndecans have prominent regulatory roles in cancer cell behaviour, the molecular basis of these effects remains mostly obscure.

Here we show that cell surface heparan sulfate proteoglycans have multiple roles in governing invasive behavior of the MDA-MB231 breast carcinoma cell line. Specifically, a key role for a syndecan-2/caveolin-2 axis is identified and characterized. Moreover, both of these components are upregulated in breast cancers, shown by tissue microarrays.

## Materials and methods

### Cell line

Human MDA-MB231 cells were grown in Dulbecco’s modified Eagle’s Media from Life Technologies (Carlsbad, CA, USA) containing 10% fetal bovine serum (FBS) at 37°C and 5% CO_2_. Cultures were screened routinely for mycoplasma contamination.

### Antibodies and reagents

The following antibodies were used: MMP14 (cat. no. ab-3644) and syndecan-1 (clone B-A38) from Abcam (Cambridge, UK); syndecan-2 (cat. no. H00006383-B04P) from Abnova (Taiwan); syndecan-2 (cat. no. LSB2981) and syndecan-4 (cat. no. LS-C150078) from LSBio (Seattle, WA, USA); caveolin-2 (D4A6) (cat. no. 8522), cadherin-11 (P707) (cat. no. 4442), MLC (cat. no. 3672) and phospho-MLC (Thr18/Ser19) (cat. no. 3674) from Cell Signaling (Beverly, MA, USA); caveolin-1 (clone 2297, cat. no. 610406) and flotillin (cat. no. 610820) from BD Biosciences (San Diego, CA, USA); p120-catenin (clone 6H11, cat. no. 339700) and transferrin receptor (clone H68.4, cat. no. 136800) from Life Technologies (Carlsbad, CA, USA); paxillin (clone 5H11, cat. no. 05–417) from Millipore (Billerica, MA, USA); β-tubulin (clone TUB2.1, cat. no. T4026) from Sigma-Aldrich (St Louis, MO, USA); Alexa Fluor-conjugated phalloidin and secondary antibodies used in immunofluorescence analysis were obtained from Life Technologies and peroxidase-conjugated secondary antibodies used in all western blotting analysis were purchased from Dako (Glostrup, Denmark).

The metalloproteinase inhibitor GM6001, DMSO, heparan sulfate from bovine kidney, chondroitin sulfate A from bovine trachea, and heparin from bovine intestinal mucosa were purchased from Sigma-Aldrich (St Louis, MO, USA). ROCK inhibitor (Y-27632) was obtained from Calbiochem (Darmstadt, Germany) and human antithrombin III was purchased from Alpha Diagnostic International (San Antonio, TX, USA).

### siRNA and DNA transfections

Cells were transfected with siRNA targeting syndecan-1 (sc-36587, Santa Cruz Biotechnology, CA, USA), syndecan-2 (sc-41045, Santa Cruz Biotechnology, CA, USA), syndecan-4 (5′-ggccgauacuucuccggaguu-3′, Qiagen, Frederick, MD, USA), MMP14 (5′-caggcaaagcugaugcagauu-3′, Qiagen), PAR-1 (5′-aaggcuacuaugccuacuacuuu-3′, Thermo Fisher Scientific, Waltham, MA, USA), caveolin-1 (siGENOME SMARTpool, Thermo Scientific), caveolin-2 (siGENOME SMARTpool, Thermo Scientific) or non-targeting siRNA (siGENOME SMARTpool, Thermo Scientific) using HiPerFect transfection reagent (Qiagen) according to the manufacturer’s instructions. Cells were analyzed 48 h after transfection.

### RNA isolation and quantitative reverse transcription-polymerase chain reaction

Total RNA was isolated using the RNeasy Micro Kit (Qiagen). Reverse transcription was performed with 1 μg of RNA by using TaqMan reverse transcription kit (Life Technologies) according to manufacturer’s protocol and quantitative PCR was performed using the Maxima SYBR Green qPCR Master Mix (Thermo Scientific) with the following primers:

syndecan-1 (forward: 5′-tactaatttgccccctgaagat-3′, reverse: 5′-caaggtgatatcttgcaaagca-3′), syndecan-2 (forward: 5′-actgttgactagtgctgctcca-3′, reverse: 5′-gggtccattttcctttctgagt-3′), syndecan-3 (forward: 5′-aagagtatcctggagcggaag-3′, reverse: 5′-agatgagcagtgtgaccaagaa-3′), syndecan-4 (forward: 5′-gtgtccaacaaggtgtcaatgt-3′, reverse: 5′-cggtacatgagcagtaggatca-3′), PAR-1 (forward: 5′-acttgatcctggccacagac-3′, reverse: 5′-acttgatcctggccacagac-3′), RPLPO (forward: 5′-ttcattgtgggagcagac-3′ reverse: 5′-cagcagtttctccagagc-3′).

### Flow cytometry analysis

Cells were harvested with cell dissociation buffer (Life Technologies), re-suspended in ice-cold sterile filtered 1% BSA/PBS and incubated at 4°C for 30 min in the presence of syndecan-1 (1:50 dilution) syndecan-2 (1:100 dilution) or syndecan-4 (1:50 dilution) antibodies or MMP14 (2 μg/ml) antibody. Cells were washed with ice-cold sterile filtered 1% BSA/PBS and further incubated with Alexa Fluor 488-conjugated secondary antibody for 30 min on ice. Following washing with ice-cold sterile filtered 1% BSA/PBS, cells were analysed on a FACSCalibur flow cytometer and data processed by using CellQuest Pro v6.0 software (Becton Dickinson, Franklin Lakes, NJ, USA). Time course experiments were organized so that the harvesting and staining procedures were synchronised.

### Fluorescence microscopy

Cells were plated on glass coverslips in complete medium (10% FBS) and after 24 h were then treated for 24 h with 20 μg/ml heparan sulfate, heparin or chondroitin sulfate, either in serum-free or serum-containing medium. Control cultures were not treated with glycosaminoglycans. Cultures were fixed in 4% paraformaldehyde and permeabilized with 0.1% Triton X-100 in PBS. After washing with PBS, free aldehydes were quenched with 0.1 M ammonium chloride, followed by blocking in 5% heat-denatured BSA. Cells were incubated with Alexa Fluor-conjugated phalloidin (1:1000) and/or primary antibody recognising cadherin-11 (1:100), p120-catenin (1:100) or paxillin (1:100) overnight at 4°C. After primary incubation, cells were washed with PBS and incubated with an appropriate fluorescent conjugated secondary antibody (1:2000) for 1 h at room temperature. Coverslips were mounted with ProLong antifade mounting medium (Life Technologies) and analysed on a Zeiss Axioplan-2 microscope (Carl Zeiss, Oberkochen, Germany). Images were processed using Metamorph (version 6.2r6) and Adobe Photoshop (version 11.0.2). ImageJ (version 1.44p) was used to quantify cell areas. For co-localization studies of syndecan-2 and caveolin-2, cells were double-stained with antibodies against syndecan-2 (1:100) and caveolin-2 (1:100), followed by Alexa-conjugated secondary antibodies. Images were acquired using a Zeiss LSM-510 confocal microscope (Carl Zeiss) equipped with a diode laser (405 nm), an argon laser (488 nm) and two helium-neon lasers (543 nm and 633 nm) and the Zen 2009 software. A 63X numerical aperture (NA) 1.4 oil-immersion Plan-Apochromat objective (Carl Zeiss) was used.

### *In vitro* invasion assay

Invasion assay were performed as previously described [27]. The membrane on the top chamber (12-well insert; pore size 8 μm, Millipore, Billerica, MA, USA) was coated with a mixture of 3 mg/ml acid-soluble type I collagen (Cellmatrix type 1-A, Nitta Gelatin, Osaka, Japan) and 10× RPMI medium (Sigma-Aldrich, St Louis, MO, USA) in a 9:1 ratio. The pH of the collagen mixture was adjusted to pH 8 with 1 M NaOH on ice. The collagen mixture was further diluted with DMEM medium to a final concentration of 2 mg/ml and incubated for 30 min at 37°C. Cells were plated on the top chamber in medium without serum and medium with serum was placed in lower chamber as a chemoattractant. The cells were incubated for 24 h and non-invasive cells were removed by cotton swab. The invasive cells were fixed, stained for DAPI and analysed on a Zeiss Axioplan-2 microscope (Carl Zeiss). Numbers of invaded cells on each whole membrane were quantified. In further control experiments, uncoated filters were used in place of collagen-coated filters.

### Collagen degradation assays

Collagen degradation assays were performed according to [27]. 12-well cell culture plates were coated with a thin layer of approx. 2.7 mg/ml PureCol™ collagen (Nutacon, Leimuiden, The Netherlands) containing 10× RPMI medium (pH 8). Plates were incubated for 1 h at 37°C to form fibrillar collagen. Cells were cultured on the fibrillar collagen for 48 h then removed by trypsin-EDTA (Life Technologies). The collagen films were fixed with 4% paraformaldehyde for 30 min, stained with Coomassie Brilliant Blue R250 and analysed on an Axiovert 135 microscope (Carl Zeiss). The clear unstained zones indicated areas of degraded collagen. Images were quantitated using Volocity 6.0.1 software.

### Western blotting and co-immunoprecipitation

Cells were lysed in sample buffer containing 62.5 mM Tris–HCl pH 6.8, 2% sodium dodecyl sulfate (SDS), 10% glycerol, 5% β-mercaptoethanol, and 0.001% bromophenol blue. For phosphorylated protein detection, cells were lysed with cold lysis buffer containing 50 mM Tris pH 7.4, 150 mM NaCl, 5 mM EDTA, 1% Triton X-100, 25 mM NaF, 2 mM NaVO_4_ and protease inhibitor cocktail (Roche, Mannheim, Germany). Cell lysates were resolved on 10% SDS-PAGE, proteins were transferred electrophoretically to PVDF membranes (Bio-Rad, USA) and blotted with the indicated antibodies. Blots were quantified using TotalLab TL100 software (Biosystematica, Devon, UK). For co-immunoprecipitation experiments, cells were lysed in ice cold buffer containing 20 mM HEPES pH7.5, 150 mM NaCl, 1% Triton-X100, 2 mM EDTA, 1 mM phenylmethylsulfonyl fluoride and protease inhibitor cocktail. The cell lysates were sheared with 25G needles and left mixing for 1 h at 4°C. The lysates were centrifuged at 13,000 rpm for 5 min at 4°C and the supernatants were pre-cleared with protein A agarose beads (Sigma-Aldrich, St Louis, MO, USA) for 1 h at 4°C. The pre-cleared lysates were incubated with caveolin-2 antibody and rabbit IgG as a control overnight at 4°C and further incubated with protein A-agarose beads for 1 h at 4°C. The beads were washed and eluted followed by electrophoresis and immunoblot analysis.

### Isolation of detergent-resistant membranes

Two confluent 15 cm dishes of MDA-MB231 cells were each scraped in 2 ml PBS on ice after 3 washes in ice cold PBS (divalent cation free). Cells were pelleted at 2°C at 900 rpm for 5 min. Some dishes were untreated, while others had been heparan sulfate (20 μg/ml) treated for the final 24 h. The two pellets were each resuspended in 0.6 ml ice-cold MNE (25 mM MES pH6.5, 150 mM NaCl, 2 mM EDTA) containing 1% Triton X-100, protease inhibitors (Roche protease inhibitor cocktail, pepstatin and 1 mM PMSF). The lysates were incubated on ice for 15 min and sheared by passage through a 25G needle 15 times, all on ice. The lysates were mixed with 0.6 ml ice-cold 80% (w/v) sucrose/MNE and divided into 2 × 1.4 ml centrifuge tubes. Each was overlaid with 0.5 ml 30% and 0.25 ml 5% sucrose/MNE in a precooled Beckman benchtop MAX-XP ultracentrifuge TLS-55 swingout rotor (Beckman Coulter, Fullerton, CA, USA). Centrifugation was carried out at 200,000 × g for 20 h at 2°C. The Triton-soluble fraction was defined as the bottom 0.2 ml of the 40% sucrose layer. The detergent-resistant membrane (DRM) fraction was clearly visible as an opaque ring at the 30-40% boundary. Each tube was fractionated into 9 × 150 μl fractions. One set from control and HS treated samples were heated with 5× sample buffer. Gels were run (10% SDS-PAGE) at 125 V. Electrotransfer to PVDF membrane was at 75 V for 90 min. The membranes were blocked in 5% milk/1% heat treated BSA in TBS for 1 h at RT, then the membranes were incubated with three antibodies simultaneously. Antibodies against flotillin were combined with those against caveolin-2 and transferrin receptor, all at 1 μg/ml in 1% milk/1% BSA/TBST at 4°C on a rocking platform. In other experiments, both antibodies against syndecan-2 or syndecan-4 were used (1:500 dilution). Membranes were washed at least 4 × over 30 min with TBST, then incubated at RT for 1 h in 1:2500 each of GAR and GAM-HRP (combined). Washing 3 × TBST over 30 min, then TBS with no Triton. Membranes were incubated with EZ-ECL (Biological Industries, Beit Haemek, Israel). Blots were visualized and photographed in the LAS4010 instrument (GE Healthcare, Freiburg, Germany).

### Immunohistochemistry

Immunostaining was performed on formalin-fixed paraffin-embedded breast tissue arrays obtained from USBiomax (Rockville, MD, USA, cat. no. BRC961, BRC962, BR486 and BRM961). The material included 24 cases of normal, reactive or benign breast tissue, 168 cases of breast cancer, from which 48 were triple negative cases and another 48 had matching lymph node metastasis or adjacent normal breast tissue. The sections were deparaffinised with xylene and rehydrated through graded alcohols into distilled water. Heat induced antigen retrieval in 0.01 M citrate buffer, pH 6.0 was performed [28]. The EnVision + System-HRP Labelled Polymer anti-Rabbit and Liquid DAB + Substrate Chromogen System (DAKO) were used for the detection. Polyclonal or monoclonal rabbit anti-human antibodies were used to detect syndecan-2 (diluted 1:50; LSBio) and caveolin-2 (diluted 1:50). Slides were counterstained with haematoxylin, dehydrated and mounted in permanent mounting medium (Eukitt quick-hardening mounting medium; Sigma-Aldrich). Slides were scanned at a 40× magnification using the NanoZoomer 2.0-HT digital slide scanner (Hamamatsu Photonics, Hamamatsu, Japan). The immunohistochemical staining was quantified and analysed. TMA cores which were either lost or fragmented were excluded from quantification analysis. Intensity and area of syndecan-2 and caveolin-2 staining were measured using BioPix iQ, version 2.1.8 (Gothenburg, Sweden) after excluding blood vessels, fat tissues and necrotic areas. Intensity per area in ln scale was plotted.

### Statistical analysis

Data are presented as standard error of mean. All western blots were quantified using TotalLAB software (Biosystematica, Devon, UK) and values are shown as ratio to controls. Two-tailed paired t-test was used to compare between groups and one-way ANOVA with Tukey’s post-hoc test was used for comparison of more than two groups. p < 0.05 was considered significant. All statistical analysis and graphs were plotted using GraphPad Prism 5 (La Jolla, CA, USA).

## Results

### Cell invasion and matrix degradation is blocked by exogenous heparan sulfate or heparin

**Figure 1:**
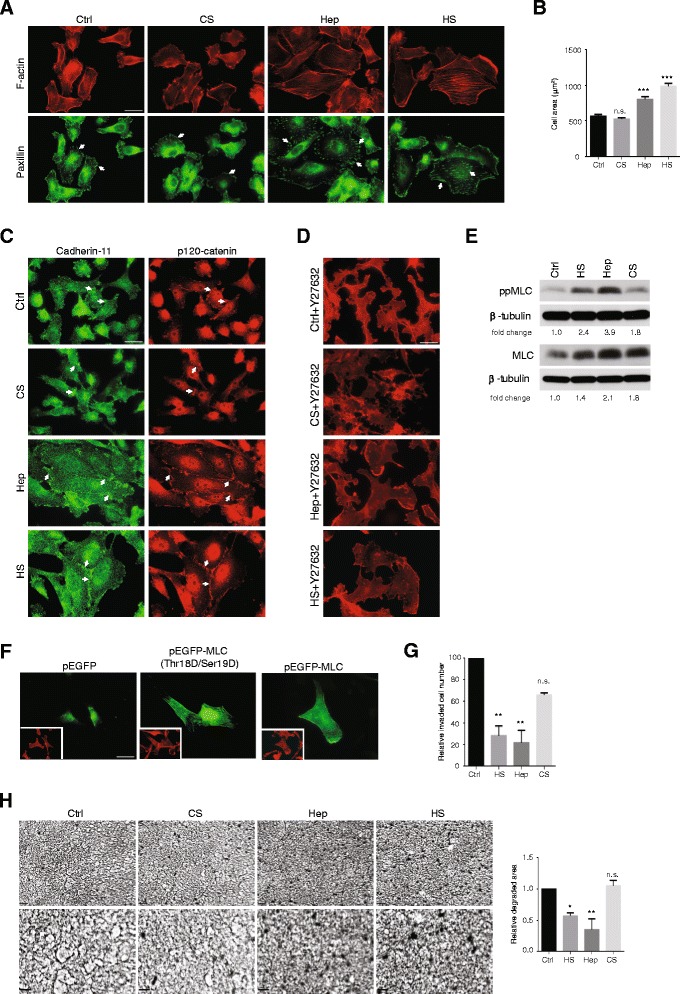
**Heparinoids promote adhesion and reduced invasive activity.** MDA-MB231 cultures were treated with 20 μg/ml heparan sulfate (HS), chondroitin sulfate (CS), heparin (Hep) or untreated (Ctrl) for 24 h after which cells were fixed and assessed for **(A)** F-actin (red) to detect microfilament bundles and paxillin (green) to detect focal adhesions (arrows), **(B)** spread cell areas (n ≥ 50 per condition), **(C)** adherens junction (arrows) components cadherin-11 (green) and p120-catenin (red). **(D)** Sensitivity of microfilament organisation to 30 μM of Rho kinase inhibitor, Y-27632 added for 30 min before fixation and staining for F-actin, Bars = 25 μm in A, C, D. **(E)** Representative western blot of diphosphorylated (Thr18/Ser19) myosin light chain (ppMLC) and total MLC from cells treated with glycosaminoglycans or untreated. Quantitation for ppMLC and total MLC levels relative to β-tubulin is shown under the blots. Similar results were obtained in three independent experiments. **(F)** F-actin organization in cells transfected with cDNAs encoding wild type MLC (pEGFP-MLC), phosphomimetic MLC (pEGFP-MLC Thr18Asp/Ser19Asp) or control vector (pEGFP). Phalloidin-stained cultures of the same areas are shown in the inserts. Bar, 25 μm. **(G)** Cells were plated onto type I collagen gel coated transwells in the presence or absence of glycosaminoglycans. After 24 h, invading cells were fixed, stained with DAPI and counted. **(H)** Cells were cultured on native type I collagen coated plates in the presence or absence of glycosaminoglycans for 48 h. After this period, cells were removed by trypsin and degraded areas were detected as clear zones by Coomassie Blue staining. Images at higher magnification are shown on the lower panels. Quantification of matrix degradation images is shown. Bar = 100 μm. Error bars = s.e.m. from three independent experiments. **p < 0.01, ***p < 0.001, n.s.: not significant. Significance was tested by one-way ANOVA with Tukey’s post-hoc test.

The malignant breast carcinoma cell line MDA-MB231 was treated with various glycosaminoglycans at low doses. On planar substrates it was noted that heparan sulfate and heparin, but not chondroitin sulfate, had a marked impact on cell morphology. Cell spreading was enhanced, average spread cell area being approximately doubled in response to heparan sulfate. The increased spreading in response to both heparin and heparan sulfate was statistically significant (Figure 1A, B). In addition, the organization of the actin cytoskeleton was also changed, with abundant microfilament bundles formed in response to the glucosaminoglycans (Figure 1A). These terminated at focal adhesions, detected by paxillin staining. Such structures are rare in untreated MDA-MB231 cells, which normally have a more rounded morphology with few microfilament bundles. Chondroitin sulfate induced no change in the actin cytoskeleton. In addition, cell-cell junctions, normally sparse in untreated cells, were increased strongly in heparin and heparan sulfate-treated cultures. In these cells, cadherin-11 (OB-cadherin) is the most abundant adherens junction adhesion receptor [29]. Staining for the cadherin-11 and p120-catenin showed extensive junction formation in response to heparan sulfate or heparin (Figure 1C), the cells showing typical epithelial characteristics, although further examination showed no indication of E-cadherin expression at junctions, with or without glycosaminoglycan treatments (not shown). Once again, chondroitin sulfate had no impact on adherens junction formation. Heparan sulfate impact on cell spreading was independent of the presence of serum in the culture medium.

Treatment of MDA-MB231 cells with Y-27632, which inhibits the Rho kinases ROCK I and II, led to total loss of microfilament bundles in cells treated with either heparin or heparan sulfate, consistent with a normal actomyosin constitution and function (Figure 1D). Consistent with this, heparan sulfate and heparin-treated cells had higher levels of Thr18/Ser19 phosphorylated myosin light chain than control or chondroitin sulfate treated cells (Figure 1E). However, further experiments showed that the cell spreading seen with heparan sulfate or heparin was more complex than simply elevated myosin light chain phosphorylation. Transfection of control cells with a cDNA encoding a mutated MLC where Thr18 and Ser19 were changed to aspartate (as a phosphomimetic [30]) led to fine microfilament bundles but no pronounced spreading (Figure 1F).

Heparin and heparan sulfate treatments markedly reduced collagen gel invasion and degradation compared to untreated controls or equivalent levels of chondroitin sulfate addition (Figure 1G, H). Since heparin is known to interact with type I collagen [31], it was important to establish whether the effects seen resulted from interactions with the matrix, or the cells themselves. In control experiments, therefore, collagen matrices were pretreated with glycosaminoglycans, and then washed before addition of mammary carcinoma cells. Under these circumstances, matrix pretreatment did not retard migration or degradation (not shown), indicating that the effects of exogenous heparin and heparan sulfate were cell-mediated.

### Inhibition of the thrombin/PAR-1 receptor signalling pathway

**Figure 2:**
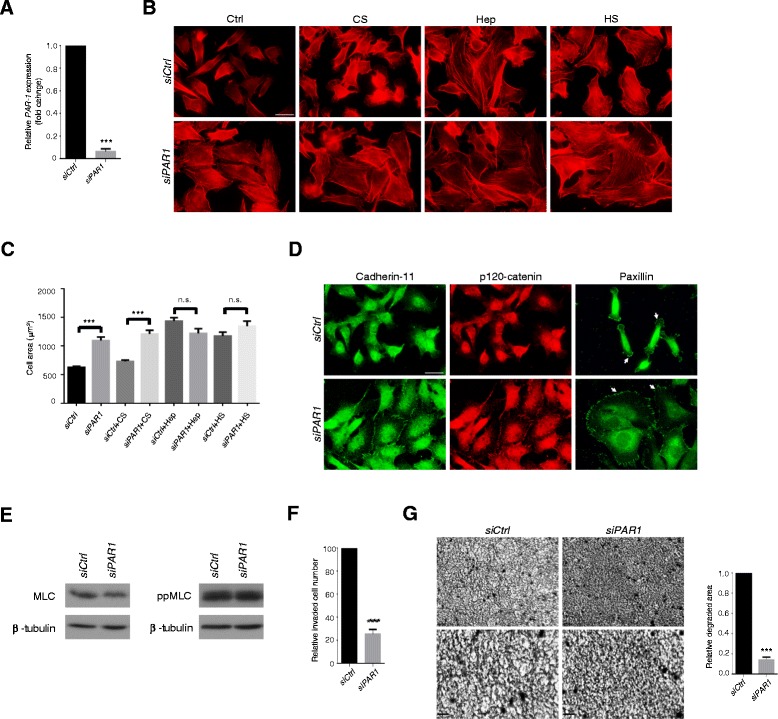
**Effects of heparinoids partly results from inhibition of thrombin.** MDA-MB231 cells were transfected with PAR-1 or control siRNA. **(A)** After 48 h, RNAs were extracted and subjected to qRT-PCR analysis to ascertain PAR-1 mRNA expression levels. siCtrl – control siRNA treatment. **(B)** PAR-1 depleted and control cells were incubated with glycosaminoglycans for 24 h, fixed and stained for F-actin. **(C)** Cell spreading induced by PAR-1 depletion and addition of glycosaminoglycans to PAR-1 depleted cells was quantified, n ≥ 50 cells per condition. **(D)** PAR-1 depleted and control cells were stained for paxillin, cadherin-11 and p120-catenin. Small focal adhesions are arrowed. **(E)**. Levels of phosphomyosin light chain (ppMLC) are unchanged by PAR-1 depeletion by siRNA. Total myosin light chain (MLC) and β-tubulin are shown. **(F)** Quantification of PAR-1 and control siRNA treated cells that invaded type I collagen gels after 24 h. **(G)** Images and quantification of type I collagen degradation by control and PAR-1 depleted cells. Images at higher magnification are shown in the lower panels. Bars = 25 μm in B and D. Bar = 100 μm in G. Error bars = s.e.m. from three independent experiments. **p < 0.01, ***p < 0.001, n.s.: not significant. Significance was tested by one-way ANOVA with Tukey’s post-hoc test **(C)** or two-tailed paired t-test **(A, F** and **G)**.

It is known that thrombin is expressed by many malignant cells, including MDA-MB231 cells [32], along with its receptor, PAR-1. Since heparin inhibits thrombin, it was possible that the effects of heparinoids on cell adhesion were related to this pathway. To test this, siRNA treatments were employed to deplete the PAR-1 receptor (Figure 2A). This led to a similar, but not identical, cellular phenotype as heparin or heparan sulfate treatment (Figure 2B). Cell spreading was markedly enhanced, but with limited formation of microfilament bundles. Spreading was not further enhanced by exposure of PAR-1 siRNA treated cells to heparin or heparan sulfate (Figure 2C). Control siRNA treated cells did not increase spreading or microfilament bundle formation, but could be induced to do so by additional heparin or heparan sulfate treatment (Figure 2B). Close examination of junction formation, however, showed that PAR-1 depletion led to modest numbers of small focal contacts or adhesions compared with heparan sulfate treatment. Adherens junction formation was not as robust either (Figure 2D). Consistent with the formation of few microfilament bundles, there was no change in myosin light chain phosphorylation in response to PAR-1 siRNA treatment (Figure 2E). Nevertheless, PAR-1 siRNA treatment significantly reduced carcinoma cell collagen gel invasion and degradation to levels similar to that achieved with heparan sulfate or heparin treatments (Figure 2F, G).

**Figure 3:**
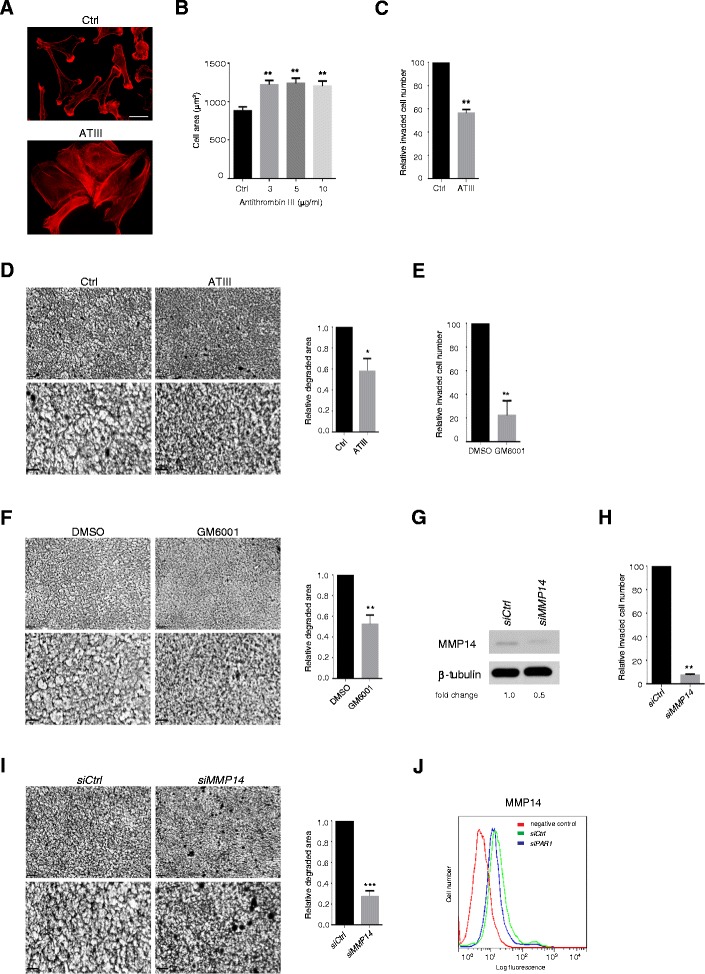
**MDA-MB231 cell collagen gel invasion requires MMP14 and is slowed by antithrombin III treatment. (A)** Various concentrations of antithrombin III (ATIII) were used to treat MDA-MB231 cells for 24 h followed by fixation and staining for F-actin. The representative images shown were cells treated with 3 μg/ml of ATIII. Bar = 25 μm. **(B)** Quantification of spread cell areas in response to ATIII treatment, n ≥ 50 cells per condition. **(C-F)** Cell invasion after 24 h **(C, E)** or degradation **(D, F)** of type I collagen gels after 48 h in the presence or absence of 3 μg/ml antithrombin III **(C, D)** or 50 μM MMP inhibitor GM6001 **(E, F)**. Higher magnification images are shown in the lower panels of D and F. Bars =100 μm. **(G)** Cell lysates were prepared 48 h after transfection with siRNA targeting MMP14 or control siRNA. Western blotting confirmed the siRNA-mediated MMP14 depletion in the treated cells. **(H, I)** MMP14 depleted cells were subjected to type I collagen cell invasion and degradation assays. Higher magnification images are shown in the lower panel (I). **(J)** Relative cell surface expression of MMP14 in PAR-1 depleted cells was assessed by flow cytometry. Error bars = s.e.m. from three independent experiments. *p < 0.05, **p < 0.01, ***p < 0.001, n.s.; not significant by two-tailed paired t-test.

In further control experiments, thrombin was inhibited by treatment with exogenous antithrombin III (ATIII) protein, which had the same effects as PAR-1 siRNA treatment (Figure 3A). Spreading was enhanced, but with modest microfilament bundle formation (Figure 3A, B). However, collagen gel invasion was reduced only by approximately 40% by ATIII, although collagen gel degradation was also reduced (Figure 3C, D). Thrombin does not cleave triple helical native collagen, so we hypothesised that the influence of PAR-1 siRNA or antithrombin III treatments were indirect, i.e. a result of enhanced cell spreading and adhesion. To confirm the role of cell-derived collagenase in collagen invasion and degradation, MDA-MB231 cells were treated with GM6001 to block matrix metalloproteinases. This almost completely prevented cell invasion and matrix degradation (Figure 3E, F). Down-regulation of MMP14 by siRNA had the same effects (Figure 3G-I), while control siRNAs had no influence on cell behavior. In particular, inhibition of cell invasion of collagen gels was near total (Figure 3H) and more effective than ATIII. This confirmed a key role for MMP14 in invasion and degradation under normal conditions. To assess whether PAR-1 depletion affected levels of cell surface MMP14, FACS analysis for the MMP after control or PAR-1 siRNA was carried out. In no case were levels of MMP14 altered compared to untreated control cultures (Figure 3J). Therefore, the impact on the thrombin/PAR-1 system of heparin and heparan sulfate appears to be separable from the role of MMP14. Moreover, heparan sulfate is an inefficient inhibitor of thrombin compared to heparin [33]. Given that thrombin inhibition only partially recapitulated the effects of exogenous heparin or heparan sulfate, we hypothesized that other pathways were also affected by the glycosaminoglycans.

### Hierarchical roles for Syndecans-2 and −4 in carcinoma cell behaviour

**Figure 4:**
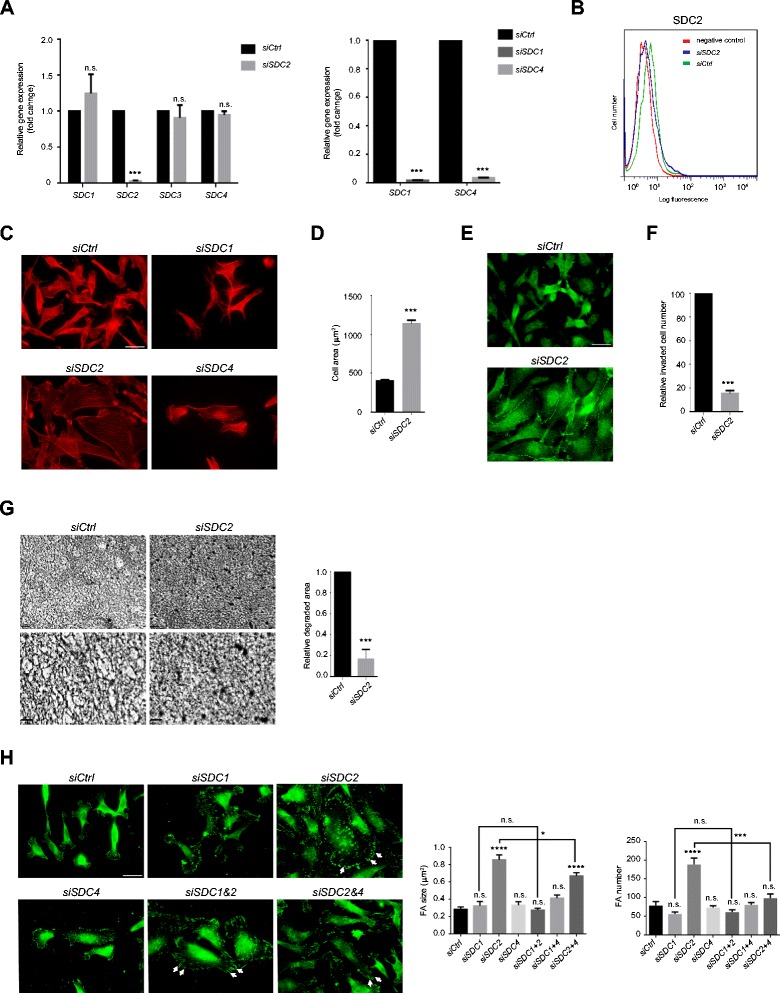
**Syndecan-2 is a regulator of MDA-MB231 cytoskeleton and behaviour. (A)** Specific knockdown of mRNA levels after syndecan-1, −2 or −4 siRNA treatments. mRNA was extracted from cells 48 h after transfection with syndecan-1, syndecan-2, syndecan-4 or control siRNAs. Levels of other syndecan mRNAs were not affected by syndecan-2 knockdown (left). **(B)** Flow cytometry analysis confirmed loss of cell surface syndecan-2 after silencing syndecan-2 with siRNA. **(C)** Syndecan-2 depleted cells were stained for F-actin, which showed extensive microfilament bundle formation. Bar = 25 μm. **(D)** Spread cell areas were increased by syndecan-2 depletion, n ≥ 50 cells per condition. **(E)** Cadherin-11 containing adherens junctions were visible after syndecan-2 depletion. Bar = 25 μm. **(F, G)** Type I collagen invasion **(F)** and degradation **(G)** were reduced in syndecan-2 depleted cells. Higher magnification images are shown on the lower panels of G. Bar = 100 μm. **(H)** Cells depleted of each syndecan individually or in combination, by siRNA treatment were stained for focal adhesions (arrows) with a paxillin antibody. Quantification of focal adhesion (FA) size and number was done by one-way ANOVA with Tukey’s post-hoc test. Bar = 25 μm. Error bars = s.e.m. from three independent experiments. ***p < 0.001, n.s.; not significant by two-tailed paired t-test.

MDA-MB231 cells express three of the four syndecan core proteins [34], as detected by qRT-PCR (Figure 4A). To determine whether exogenous heparinoids were competing with these heparan sulfate bearing molecules, each syndecan was knocked down by siRNA, either singly or in combination. Knockdown was confirmed by qRT-PCR and FACS analysis (Figure 4A, B). Striking results were obtained by syndecan-2 depletion. MDA-MB231 cells acquired a spread morphology, with increased microfilament bundles, cadherin-11 containing adherens junctions and significantly enhanced focal adhesions (Figure 4C-E, H). Carcinoma cell invasion and degradation of type I collagen matrices were commensurately inhibited (Figure 4F, G). This suggested that heparin or heparan sulfate could compete with syndecan-2 to bring about actin cytoskeletal changes. It was confirmed that siRNA for syndecan-2 had no influence on mRNA levels for the other syndecans (Figure 4A). Control experiments with uncoated, rather than collagen coated transwells showed that cell migration was not inhibited by syndecan-2 siRNA treatment (not shown). This suggests that the absence of syndecan-2 specifically decreases extracellular matrix-mediated migration.

No effects on cell adhesion or junction formation were noted where syndecan-1 or syndecan-4 were depleted on their own. Moreover, no additional effects were noted where syndecan-1 was depleted in combination with syndecan-2 knockdown (Figure 4H). However, double depletion of syndecan-2 and −4 had one clearly observable effect, a decrease in the size and number of focal adhesions, compared with syndecan-2 knockdown alone (Figure 4H). These data are consistent with previously published roles for syndecan-4 in focal adhesion assembly [35]. As a whole the data suggest that syndecan-2 in breast carcinoma plays a major regulatory role in maintaining the invasive phenotype and appears to suppress the focal adhesion promoting role of syndecan-4.

**Figure 5:**
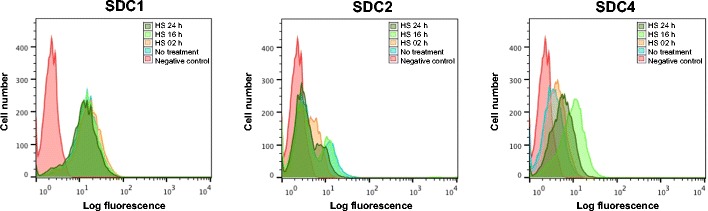
**Heparan sulfate treatment causes loss of syndecan-2 and gain of syndecan-4 on the cell surface.** MDA-MB231 cells were analysed at 2, 16 and 24 h after treatment with 20 μg/ml heparan sulfate for cell surface levels of syndecans-1, −2 and −4. Control cells were untreated. FACS analysis showed decreased syndecan-2 to near background, while levels of syndecan-4 increased. Syndecan-1 levels were unchanged throughout the treatment.

Further experiments examined the impact of heparin and heparan sulfate on cell surface levels of syndecans-1, −2 and −4 (Figure 5). Over 24 h, FACS analysis revealed that levels of syndecan-2 reduced to near background, while levels of syndecan-4 increased. Syndecan-1 levels were unchanged throughout. These data are consistent with the impact of syndecan-2 siRNA which leads to loss of the proteoglycan and an effect of syndecan-4 on focal adhesion assembly, which is enhanced by heparan sulfate treatment (Figure 1A). At the same time, knockdown of syndecan-2 had no effect on cell surface PAR-1 levels (not shown), indicating that the effect of syndecan-2 on cell adhesion was independent of PAR-1.

### Caveolin-2 regulates invasive phenotype of MDA cells

**Figure 6:**
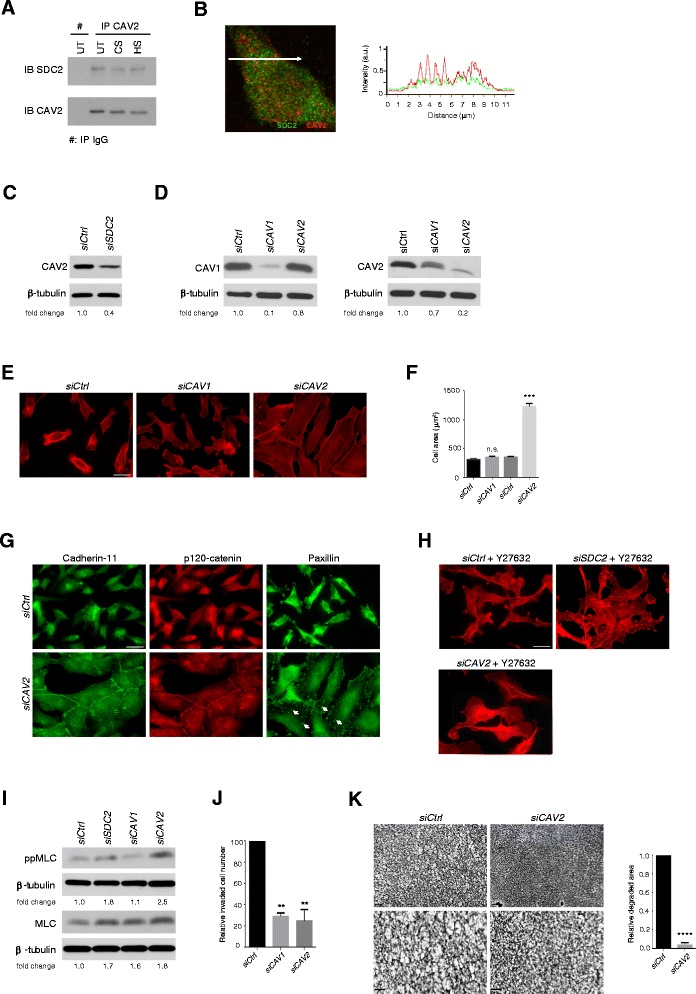
**Caveolin-2 interacts with syndecan-2 and regulates cell adhesion in MDA-MB231 cells. (A)** Syndecan-2, but not syndecan-4, was co-immunoprecipitated with caveolin-2 from cell lysates. Co-immunoprecipitation was not affected by ectopic glycosaminoglycan treatment of the cells (UT; untreated, CS; chondroitin sulfate, HS; heparan sulfate). **(B)** Confocal laser scanning microscopy and profile of the line scanning (white arrow on image) confirmed partial co-localization of syndecan-2 (green) and caveolin-2 (red). **(C)** Caveolin-2 levels were reduced where syndecan-2 was depleted by siRNA, compared with control siRNA. **(D)** Western blotting verified the knockdown efficiency of siRNA targeting caveolin-1 and −2 compared to control siRNA. Downregulation of caveolin-1 reduced the expression of caveolin-2 by around 30%, but knockdown of caveolin-2 had no impact on caveolin-1 levels. **(E)** F-actin containing microfilament bundles were abundant after caveolin-2, but not caveolin-1 depletion. Bar = 25 μm. **(F)** Spread cell areas were measured in caveolin-2 depleted cells and control cells, n ≥ 50 per condition. **(G)** Adherens junctions and focal adhesions (arrows) were characteristic of caveolin-2 depleted cells, shown by cadherin-11 and p120-catenin, or paxillin distributions. Bar = 25 μm. **(H)** Microfilament bundles formed in response to either syndecan-2 or caveolin-2 knockdown were sensitive to 30 μM Rho kinase inhibitor, Y-27632. Bar = 25 μm. **(I)** Diphosphorylated myosin light chain (ppMLC; Thr18/Ser19) was enhanced in both syndecan-2 and caveolin-2 depleted cells. Densitometry analysis of western blots for ppMLC and total MLC were normalised to β-tubulin. Similar results were obtained in three independent experiments. **(J, K)** Caveolin-2 depleted cells had reduced ability to invade or degrade type I collagen gels. Higher magnification images are shown in the lower panels. Caveolin-1 depletion also reduced collagen gel invasion. Bar = 100 μm. Error bars = s.e.m. **p < 0.01, ***p < 0.001, n.s.; not significant by two-tailed paired t-test.

Little is understood regarding the signalling function of syndecan-2. Along with other syndecans, it can bind PDZ domain proteins through its conserved C2 cytoplasmic region. However, binding partners for its unique V region are unknown. There is, however, one report that syndecan-2 can interact, directly or indirectly, with caveolin-2 [36]. Indeed, caveolin-2 could be shown to form a complex with syndecan-2 in MDA-MB231 cells, which is consistent with the previous study (Figure 6A). By confocal microscopy, partial colocalization of syndecan-2 and caveolin-2 was apparent (Figure 6B). However, caveolin-2 did not associate with syndecan-4. In addition, caveolin-2 protein level was reduced upon syndecan-2 depletion (Figure 6C). Therefore, in further experiments, caveolin-1 and −2 were separately reduced by siRNA treatments. It was confirmed that specific knockdown of caveolin−2 had no effect on protein levels of the alternate caveolin (Figure 6D). However, knockdown of caveolin-1 resulted in approximately 30% reduction in caveolin-2. In terms of cell adhesion, the results were strikingly different for knockdown of the two proteins. Reductions in caveolin-1 had no impact on cell spreading or cytoskeletal organization, although invasion of collagen gels was reduced (Figure 6E, F, J). Depletion of caveolin-2, however, recapitulated the effects of adding exogenous heparin or heparan sulfate, or syndecan-2 depletion. The breast carcinoma cells increased spreading, microfilament bundle and focal adhesion assembly, and increased the extent of cadherin 11-mediated adherens junctions (Figure 6E-G). Concomitantly, collagen degradation and invasion were much reduced (Figure 6J, K).

To investigate whether the formation of microfilament bundles was Rho kinase dependent, cells after syndecan-2 or caveolin-2 depletion were treated with the ROCK inhibitor, Y-27632. Similar to heparan sulfate or heparin treatment, Y-27632 treatment inhibited microfilament bundle formation triggered by syndecan-2 or caveolin-2 depletion (Figure 6H). In addition, synedecan-2 or caveolin-2 depleted cells, but not caveolin-1 depleted cells, had higher levels of Thr18/Ser19 phosphorylated myosin light chain compared to controls (Figure 6I). The data suggest that microfilament bundle formation observed in the absence of syndecan-2 or caveolin-2 was Rho kinase-dependent.

**Figure 7:**
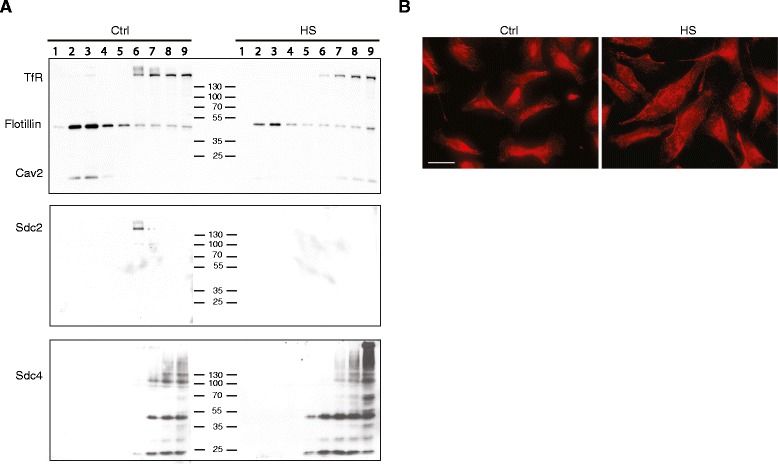
**Exogenous heparan sulfate influences subcellular localisation of caveolin-2. (A)** Detergent-resistant membrane (DRM) preparations were made from MDA-MB231 lysates, some treated with 20 μg/ml heparan sulfate for 24 h. Nine fractions were collected from each centrifuge tube and western blotted for flotillin as a marker of DRM pools, transferrin receptor as a marker of detergent soluble membrane proteins (non-DRM), and caveolin-2. The caveolin-2 is almost exclusively in the DRM pool of control cells, while it is displaced to the non-DRM pool when cells are treated with heparan sulfate. Syndecan-2 is reduced after heparan sulfate treatment **(B)** Caveolin-2 staining of untreated (Ctrl) MDA-MB231 cells and cells treated with 20 μg/ml heparan sulfate (HS) for 24 h before fixation. While the HS-treated cells are more spread than control cells, there is no obvious alteration in the localisation of caveolin-2. Bar = 25 μm.

Caveolins are involved in endocytic events, and are key components of caveolae. While caveolin−1 and −2 interact, specific functions of caveolin-2 are recognized. To explore this further, detergent-resistant membrane (DRM) preparations were made from MDA-MB231 cells, using published methods [37]. The detection of flotillin, known to be present in these preparations served as a marker of the DRM pool. Transferrin receptor was detected as a component known not to associate with these membrane entities [38], Analysis of the fractions obtained from sucrose density gradient ultracentrifugation showed, as expected, that a major pool of flotillin could be detected in the low density fractions, but also in other parts of the gradient. Transferrin receptor was only present in the bottom fractions (Figure 7A). Caveolin-2 was exclusively present in the low density DRM pool but was lost from the low density fractions where the cells were treated for 24 h before lysis with 20 μg/ml heparan sulfate, shown to induce spreading, adhesion and reduced invasive behavior. Therefore, a key finding is that caveolin-2 was relocated as a membrane component from a triton-resistant (DRM) pool to a triton-soluble pool in response to heparan sulfate treatment. At the light microscopic level, however, no clear change in the distribution of this protein could be seen under identical treatment conditions (Figure 7B). Caveolin-1, on the other hand was not exclusive to the DRM pool in control cells, and not significantly altered in buoyant density when cells were glucosaminoglycan-treated (not shown). Syndecan-2 was located in a pool midway through the gradient and was undetectable in membrane fractions after heparan sulfate treatment, indicative of decreased abundance. Syndecan-4, on the other hand, was not reduced, but present in more of lower fractions after heparan sulfate treatment. These data are consistent with the FACS analysis (Figure 5), showing decreased syndecan-2 and increased syndecan-4 on the cell surface after heparan sulfate treatment,

### Syndecan-2 expression is elevated in metastatic breast tumour

**Figure 8:**
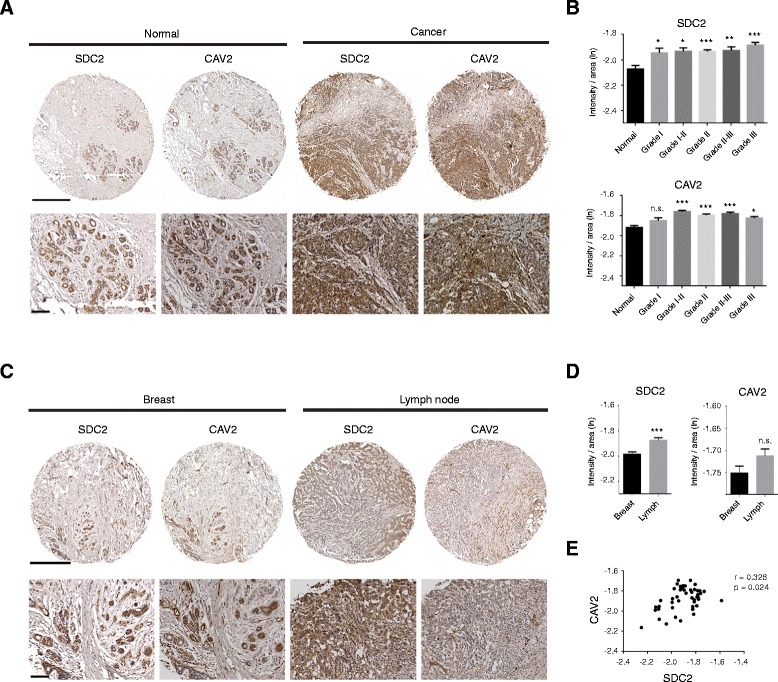
**Syndecan-2 expression correlates with metastatic status in patient. (A)** Normal breast tissues (Normal) and breast cancer patient tissues (Cancer) from tissue microarrays were stained for syndecan-2 or caveolin-2 on serial sections. Magnified images are shown in lower panels, bars = 100 μm. **(B)** Intensity per area for syndecan-2 or caveolin-2 staining was quantified for each core in tissue microarray to compare normal breast tissue and patient breast tissue of different tumour grades, n = 161 for syndecan-2 and n = 165 for caveolin-2. Bar graph was plotted in ln scale. **(C)** Breast tissue from patients diagnosed with Grade II carcinoma and metastatic lymph node tissue from the same patient were stained for syndecan-2 and caveolin-2. Higher magnification images are shown in lower panels, bars = 100 μm. **(D)** Quantification data of intensity per area of syndecan-2 or caveolin-2 staining from **(C)**, n = 26 for syndecan-2 cases and n = 28 for caveolin-2 cases. Bar graph was plotted in ln scale. Error bars = s.e.m. *p < 0.05, **p < 0.01, ***p < 0.001, n.s.: not significant, tested by one-way ANOVA with Bonferroni post-hoc test. **(E)** Levels of syndecan-2 and caveolin-2 staining from serial sections of the 47 triple negative breast carcinoma cases were significantly correlated. r: Spearman’s correlation coefficient = 0.328, p = 0.024.

To determine whether syndecan-2 and caveolin-2 expression correlates with disease and clinical outcome in breast cancer patients, we performed immunohistochemical analysis of human breast tissue microarrays. Syndecan-2 was examined in 161 cases while caveolin-2 was examined in 165 cases. Wherever possible, serial sections were stained for the two proteins. In normal tissue, cytoplasmic syndecan-2 expression was observed in myoepithelial cells around the ducts, which also co-expressed caveolin-2 (Figure 8A), suggesting that syndecan-2 and caveolin-2 may also cooperate in signal transduction *in vivo*. In tumor cells, syndecan-2 staining was cytoplasmically distributed but we also noted that some displayed nuclear staining. More importantly, syndecan-2 and caveolin-2 staining were significantly elevated in cancer patient samples compared to normal tissues regardless of tumor grade (Figure 8B). We also performed immunohistochemical analysis to compare expression of these two proteins in a small number of matching primary breast tumors and lymph nodes from patients diagnosed with Grade II status. A significant increase in syndecan-2 staining intensity was observed in lymph nodes compared to primary tumors, suggesting that syndecan-2 associates with metastasis during the course of cancer progression (Figure 8C, D). In contrast, we did not detect significantly increased caveolin-2 staining in lymph nodes compared to primary tumors. A closer examination of 47 triple negative breast cancer cases showed a significant correlation in the expression of syndecan-2 and caveolin-2 (Figure 8E). These data demonstrated that syndecan-2 together with caveolin-2 are highly expressed in breast cancers and that they can combine to promote an invasive phenotype *in vitro*.

## Discussion

Heparin and heparan sulfate are frequently used as exogenous competitors of cell surface proteoglycan-mediated events. These glycosaminoglycans potently influenced the behavior and cytoskeletal organization of the invasive MDA-MB231 cell line, which is triple negative (i.e. lacking estrogen, progesterone and Her2Neu/ErbB2 receptors). Their effects appeared to be due to inhibition of two major signaling pathways, thrombin interactions with PAR-1 receptor, and that mediated by a syndecan-2/caveolin-2 pathway.

Thrombin is a serine protease with key roles in the coagulation cascade and can be inhibited by heparin [39]. Thrombin signaling is mediated through a family of small family of G-protein-coupled protease activated receptors (PARs). Of the four PAR family members, PAR-1, is the prototypical member and has been identified as a potent oncogene based on its ability to stimulate focus formation and induce NIH3T3 cell transformation [40]. In addition to their normal physiological roles in vascular biology, there is substantial evidence for aberrant expression of thrombin and PAR-1 in several cancers, including breast cancer [32,41-43], melanoma [44,45] and prostate cancer [46,47]. In breast cancer, increased PAR-1 expression was closely associated with the invasive capacity of primary breast tissue specimens and breast carcinoma cell lines [32,41,43,48,49], suggesting PAR-1 has a critical role in tumor progression. We have demonstrated that heparan sulfate and heparin may inhibit PAR-1 activation by inhibiting thrombin, leading to cell spreading and attenuated invasiveness of MDA-MB231 cells. However, PAR-1 depletion did not alter the cell surface expression levels of the major metalloproteinase, MMP14, which was confirmed here to be essential for collagen invasion and degradation.

We also show that a heparan sulfate proteoglycan, syndecan-2 is a regulator of breast carcinoma invasiveness. Cell invasion and degradation of type I collagen were remarkably inhibited after syndecan-2 depletion. Furthermore, reduction in cell invasiveness was accompanied by changes in actin cytoskeletal organization and cell-cell adhesion, where cell spreading, microfilament bundles, focal adhesions, and cadherin-11 containing adherens junctions were all enhanced. Thus depletion of syndecan-2 reveals a mechanism by which it controls the invasiveness of MDA-MB231 cells. Consistent with this heparan sulfate treatment was shown by FACS analysis to lead to specific syndecan-2 loss from the cell surface, while levels of syndecan-4 increased and syndecan-1 was unchanged. Focal adhesion and microfilament bundle formation in response to loss of syndecan-2 was shown to depend on the presence of syndecan-4. Very recent work suggests that this mechanism involves p190ARhoGAP, which associates with syndecan-4 and active β1 integrin at the cell margins in the absence of syndecan-2 [50]. RhoGAP promotes conversion of GTP-Rho to the inactive GDP-bound form but is inactivated by Src-mediated tyrosine phosphorylation. Previous work has demonstrated the importance of syndecan-4 regulation of p190RhoGAP distribution [51] and our data correspondingly show that Rho kinases (ROCKs) and their downstream target, myosin light chain, are required for focal adhesion and microfilament bundle formation in MDA-MB231 cells upon heparan sulfate addition or syndecan-2 depletion.

A very recent study illustrated that syndecan-2 depletion in MDA-MB231 derived 1833 cell line with enhanced bone tropism led to inhibition of breast tumor growth and metastasis to bone in a mouse xenograft model, in part through enhanced apoptosis [52]. No effects on junctions or actin cytoskeleton were reported in this study. However, we did not observe changes in growth or cleaved caspase-3 staining after syndecan-2 depletion (data not shown), indicating that the parental MDA-MB231 cells and MDA-MB231 derived 1833 cells might signal through different pathways to regulate invasive behaviour. The current data strongly suggest that syndecan-2 is a tumor promoter by regulating cytoskeletal organization and tumorigenic activity in breast cancer cells.

In MDA-MB231 cells, syndecan-2 combines with caveolin-2 to promote an aggressive phenotype. Caveolin is the major structural component of plasma membrane microdomains that play numerous pivotal roles in intracellular trafficking and signal transduction [53,54]. The MDA-MB231 cell line is known to carry a KRas G13D mutation [55]. Upon oncogenic Ras transformation in BalbC/3 T3 cells, syndecan-2 has been shown to form a complex with caveolin-2 [36], which was also shown here. The interaction of caveolin-2 with syndecan-2 is specific, since caveolin-2 did not form a complex with syndecan-4. This interaction and its consequences deserve further attention. Strikingly, caveolin-2 depletion yielded the same cell behavior and cytoskeletal organization as syndecan-2 depletion, with increased cell spreading, microfilament bundles, larger and more numerous focal adhesions and diminished type I collagen invasion and degradation. Unlike caveolin-2, caveolin-1 depletion did not give rise to an altered phenotype, though cell invasion was reduced in these cells. Caveolin-1, but not caveolin-2 has been identified as being important for the formation and activity of invadopodia [56]. Therefore, reduced cell invasion in caveolin-1 depleted cells may result from impaired invadopodia function. Caveolin-2, on the other hand, might stimulate cell invasiveness through different mechanisms than caveolin-1, which awaits further elucidation.

In breast carcinoma, caveolin-2 expression is frequently upregulated and correlates with poor prognostic status [57,58]. However, there is no previous evidence for involvement of a syndecan-2/caveolin-2 axis in breast tumor cell metastasis. Our patient tissue microarray data showed that syndecan-2 expression and caveolin-2 were significantly increased in breast cancer patients regardless of tumor grade. More importantly, syndecan-2 expression was upregulated in lymph node samples compared directly with the primary tumours and in triple negative cases, expression of syndecan-2 and caveolin-2 were correlated.

Syndecan-2 has remained very elusive with regard to its signaling ability. The syndecan-2/caveolin-2 relationship may provide an important insight into regulation of a specific membrane microdomain in tumour, and perhaps untransformed cells. Previous work has shown that syndecan-1 upregulation in breast carcinoma is associated with poor prognosis, particularly when present in the stroma [21,59]. Recent data also suggests that syndecan-2, normally considered a mesenchymal proteoglycan, is upregulated and a potential target in colon carcinoma [18]. It was also noted as upregulated by gene expression profiling in breast cancer [60] but insight into its function has been lacking. Given the importance of syndecans in breast, colon and other cancers such as myeloma [61] their role on the cell surface is increasingly important to understand. The fact that exogenous heparan sulfate and heparin inhibit two pathways that support an invasive phenotype suggests that cell surface proteoglycans are a promising area for development of reagents and understanding of breast carcinoma invasion and metastasis.
